# Advances in Textile Structural Composites II

**DOI:** 10.3390/polym17121603

**Published:** 2025-06-09

**Authors:** Rajesh Kumar Mishra

**Affiliations:** Department of Material Science and Manufacturing Technology, Faculty of Engineering, Czech University of Life Sciences Prague, 16500 Prague, Czech Republic; mishrar@tf.czu.cz

Textile structural composites are increasingly being recognized in engineering fields due to their exceptional mechanical attributes, lightweight nature, and improved durability when compared to conventional materials. These composites consist of textile structures from glass, carbon, aramid, or natural fibers, that are integrated within a polymer, metal, or ceramic matrix, leading to outstanding strength-to-weight ratios, resistance to corrosion, and versatility in design. The mechanical properties of such composites are influenced by factors such as the type of textile reinforcement, fiber orientation, and interfacial bonding, which affect characteristics like tensile strength, impact resistance, and fatigue performance [1]. These qualities are further enhanced by hybrid composites, which blend various fabric types to provide increased structural efficiency. The final composite characteristics and structural integrity are affected by a number of production processes, including pultrusion, vacuum-assisted resin infusion (VARI), resin transfer molding (RTM), manual lay-up, and additive manufacturing. By maximizing resource use and cutting waste, emerging fabrication techniques like 3D printing and automated fiber placement are completely changing the business. Textile-based composites are used in several engineering applications in the sports equipment, automotive, marine, aerospace, and civil infrastructure sectors. Their high-performance qualities help reduce weight and improve durability and energy efficiency [2].

They are utilized in the automobile industry for body panels, chassis components, and crash-resistant structures for enhanced vehicle performance and safety, and in aerospace for fuselage panels, wing structures, and interior components. Boat hulls and offshore constructions are examples of marine applications that take advantage of their resilience to severe weather conditions. To ensure lifespan and improved performance, civil engineering incorporates fiber-reinforced composites (FRCs) into bridge decks, structural rehabilitation, and seismic retrofitting [3]. The high strength-to-weight ratio, corrosion resistance, ease of fabrication, and improved design flexibility are just a few of the many benefits of FRCs. However, issues like moisture absorption, recycling difficulty, environmental effect, and high initial prices continue to stand in the way of wider implementation. To overcome these constraints, efforts are being made to improve material efficiency and environmental sustainability through the development of sustainable bio-based composites, sophisticated recycling methods, and computer modeling. FRCs’ role in high-performance engineering applications will grow as a result of their ongoing development, which is being fueled by breakthroughs in material science and creative production techniques [4].

In order to guarantee their widespread integration into next-generation engineering solutions, this Special Issue included futuristic research directions and possible advancements while providing a thorough overview of textile structural composites, examining their performance, fabrication processes, applications, benefits, and drawbacks.

In addition to their structural and mechanical benefits, textile-reinforced composites contribute to the creation of smart and multifunctional materials, which integrate additional characteristics such as self-healing, impact sensing, and temperature management. These composites can be modified with nanoparticles, conductive fibers, and embedded sensors to improve damage detection and predictive maintenance in important structures [5]. Industries like defense and biomedical engineering are studying these composites for ballistic protection, prostheses, and bio-inspired applications where durability and adaptability are critical [6]. The future of FRCs seems bright as long as research into sustainable reinforcements, artificial intelligence-driven material design, and computational modeling continues [7]. To standardize testing procedures, increase their manufacturability, and guarantee their wider acceptance across engineering domains, increased cooperation between academic institutions, business, and regulatory agencies is crucial. Engineers can unleash the potential of next-generation materials and create innovations that redefine structural performance and material efficiency across industries by incorporating sustainability and digital advances into the creation of textile-reinforced composites [8].

This Special Issue is the subsequent addition to a previous Special Issue on the same topic and continues to provide further insights into advanced textile geometrical structures used as reinforcement in composites. In this Special Issue, the computational and geometrical models that can effectively predict the failure occurring in the textile reinforcement composite structures were discussed.

The applications and potential of statistical evaluation in textile-based composites were elaborated. The valorization and utilization of textile wastes for creating value-added composites was given utmost importance for sustainable product development. Bio-based materials and their importance for textile structures as well as composites were further discussed. Unconventional and modern application areas, e.g., biomedical, electronic gadgets, communication, sensors, etc., were explored.

The Editor is thankful to all the contributors and editorial staff for preparing this Special Issue successfully and effectively. These specialized contributions will lead to new methods of research and development in these emerging areas of sustainable composite product development.
